# Atmospheric Organic Nitrogen Deposition in Strategic Water Sources of China after COVID-19 Lockdown

**DOI:** 10.3390/ijerph19052734

**Published:** 2022-02-26

**Authors:** Yixuan Yang, Tongqian Zhao, Huazhe Jiao, Li Wu, Chunyan Xiao, Xiaoming Guo, Chao Jin

**Affiliations:** 1 Institute of Resources and Environment, Henan Polytechnic University, Jiaozuo 454000, China; yangyixuan@hpu.edu.cn (Y.Y.); wuli@hpu.edu.cn (L.W.); xiaochunyan@hpu.edu.cn (C.X.); guoxiaoming@hpu.edu.cn (X.G.); jinc1126@163.com (C.J.); 2 School of Civil Engineering, Henan Polytechnic University, Jiaozuo 454003, China; jiaohuazhe@126.com

**Keywords:** atmospheric nitrogen deposition, strategic water sources, COVID-19 epidemic lockdown, human activities, organic nitrogen, urea nitrogen

## Abstract

Atmospheric nitrogen deposition (AND) may lead to water acidification and eutrophication. In the five months after December 2019, China took strict isolation and COVID-19 prevention measures, thereby causing lockdowns for approximately 1.4 billion people. The Danjiangkou Reservoir refers to the water source in the middle route of South-to-North Water Diversion Project in China, where the AND has increased significantly; thus, the human activities during the COVID-19 period is a unique case to study the influence of AND to water quality. This work monitored the AND distribution around the Danjiangkou Reservoir, including agricultural, urban, traffic, yard, and forest areas. After lockdown, the DTN, DON, and Urea-N were 1.99 kg · hm^−2^ · month^−1^, 0.80 kg · hm^−2^ · month^−1^, and 0.15 kg · hm^−2^ · month^−1^, respectively. The detected values for DTN, DON, and Urea-N in the lockdown period decreased by 9.6%, 30.4%, and 28.97%, respectively, compared to 2019. The reduction in human activities is the reason for the decrease. The urban travel intensity in Nanyang city reduced from 6 to 1 during the lockdown period; the 3 million population which should normally travel out from city were in isolation at home before May. The fertilization action to wheat and orange were also delayed.

## 1. Introduction

**Highlight**

- The DTN, DON and Urea-N in AND decreased during epidemic lockdown.
- The decrease can be explained by traffic stopping and fertilization being delayed.
- The nitrogen deposition flux in the Danjiangkou area is at a medium level in China.

Solid waste and air pollution are important sources of pollution caused by human activities to the natural environment [1,2,3]. Human activities have an important impact on atmospheric nitrogen deposition (AND). The worldwide COVID-19 outbreak in late 2019 has had a very serious impact on human activities. The regularity of dissolved organic nitrogen (DON) deposition in strategic water sources before and after COVID-19 has become a very meaningful research topic.

On 30 January 2020, China implemented strict quarantine measures, and the entire country was under comprehensive and strict control, i.e., the country was in lockdown. At the end of April 2020, the country gradually entered the state of unsealing, wherein personnel began to move, and industrial and agricultural production resumed in an all-round way. For three months, approximately 1.4 billion Chinese people and their related activities stopped, which is also a unique example in human history [4]. The impact of COVID-19 lockdown on the environment is an excellent case to be explored.

After the COVID-19 outbreak, global carbon emissions were reduced by 2.5 billion tons. The lockdown in various countries have led to the stopping of factories and vehicles, and the air quality has improved significantly [5,6,7].

Since the 20th century, in addition to the carbon emission, human activities have also discharged nitrogen-containing compounds into the atmosphere. Asia (China, India), Western Europe, and North America have become the three largest nitrogen deposition concentration areas [8]. The significant increase in AND leads to the acidification, eutrophication, and biodiversity reduction in soil, water and ecosystems [9], which seriously threatens the water and terrestrial ecosystems [10]. At the beginning of 2020, the tropospheric NO_2_ column in eastern China decreased by 65% compared with the same period in 2019, mainly caused by the change of fossil fuel combustion after the epidemic response period [11].

DON is an important component of nitrogen deposition, approximately 15–30% of total deposition flux. The research on DON can be traced back to the early 20th century. The Lausanne experimental station in the UK has researched the total DON in rainwater for several years. The results show that the maximum concentration of DON in rainwater is 2.50 μmol/L, average 1.4 μmol/L, and its contribution to total nitrogen is roughly 25% [12]. In the study of AND in Rio de Janeiro, Brazil, de et al. [13] found that the variation range of DON concentration in the mixed deposition of urban areas is 15.7–50.6 μmol/L, accounting for 32–56% of DTN. The concentration variation range of forest areas is 10.1–10.9 μmol/L, accounting for 26–32% of DTN. Wang et al. [14] found that the proportion of DON in the DTN in the air increased from 30 to 80% after the dust event. The global atmospheric organic nitrogen deposition is estimated to be 10–50 Tg · a^−1^.

To further understand the impact of DON on the ecosystem, the composition of DON needs to be analyzed. Urea is an organic nitrogen compound often detected in AND. Urea is easily absorbed and utilized by organisms and is one of the main factors causing water eutrophication [15]. In phosphorus-rich lakes, urea can promote the growth of some harmful algae and the production of toxins, thereby posing a threat to the ecosystem and human health.

The Danjiangkou reservoir is located between Hubei and Henan province. It is the water source of the middle route of the South-to-North Water Diversion Project in China. The South-to-North Water Diversion Project is a major water conservancy project to alleviate the drought and water shortage in northern China and to ensure the safety of the water supply [16]. The water quality of the Danjiangkou reservoir is directly related to the water safety of more than 20 large cities, such as Beijing and Tianjin [17]. According to the data of Henan annual report on environmental quality, the total nitrogen content in the reservoir area is 1.0–1.5 mg/L, exceeding the class III water quality standard of surface water (applicable to the surface water source of centralized domestic and drinking water), which has become the main factor that affects the water quality safety of the Danjiangkou reservoir [18].

Studies on nitrogen deposition in the Danjiangkou reservoir are few [19]. Nitrogen deposition is affected by human activities. Therefore, this work studies the impact of human activities on the AND before and after the COVID-19 lockdown in the Xichuan reservoir area in the Danjiangkou Reservoir.

As shown in literature [20], the government carried on Initial Progress in Containing the Virus, January 20, the citied were locked down. The status was changed to Ongoing Prevention and Control from 29 April 2020. The lockdown finished in early May, 2020 [21].

So, the periods of fighting COVID-19 can be defined as three stages. The early epidemic period is before January 2020, in which the cities were about to lockdown [22]. The middle epidemic period is February–April 2020, in which, the cities were locked down, and people were in isolation at home. The later epidemic period is after May 2020, when the population could move around [23,24].

In this work, the nitrogen deposition around the reservoir was detected from January 2019 to May 2020. The DTN, DON and Urea-N in this period will be compared with the early epidemic period, middle epidemic period and after the epidemic lockdown. Furthermore, the nitrogen deposition data will be compared with the same period in 2019 to investigate the impact of human activity on the nitrogen deposition.

## 2. Materials and Methods

### 2.1. Sampling Locations

**Figure 1:**
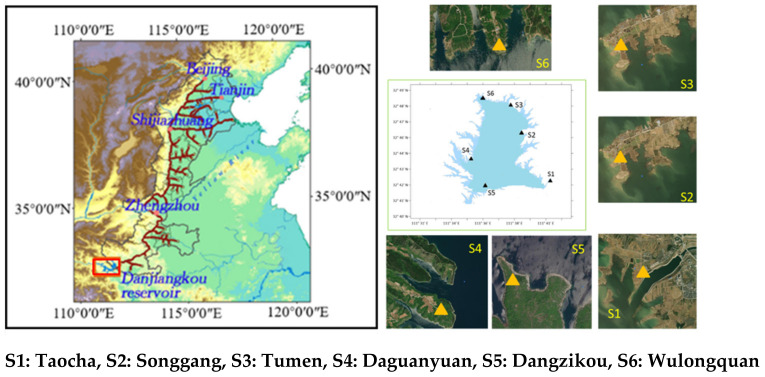
Sampling point position and automatic sampler.

Six sampling stations are set around Xichuan reservoir area, as shown in Figure 1. AND samples are collected to determine DTN, dissolved NH_4_^+—^N, and NO_3_—N.

Taocha (S1) station is located around the canal head with large surrounding population and the developed traffic status. The location is defined as urban area, where the main pollution sources are daily waste and road traffic pollution.

Songgang (S2) station is located at the wharf. The location is defined as traffic area, where the main pollution source is traffic and waste pollution.

Tumen (S3) station is close to wheat and corn farmland. The location is defined as agricultural area, where pesticides and fertilizers are sprayed. There are pollution sources of livestock manure and waste.

Daguanyuan (S4) station is a scenic spot. There is a large flow of people and vehicles on holidays. Its main pollution sources are traffic and waste pollution. The location can be defined as traffic area.

**Figure 2:**
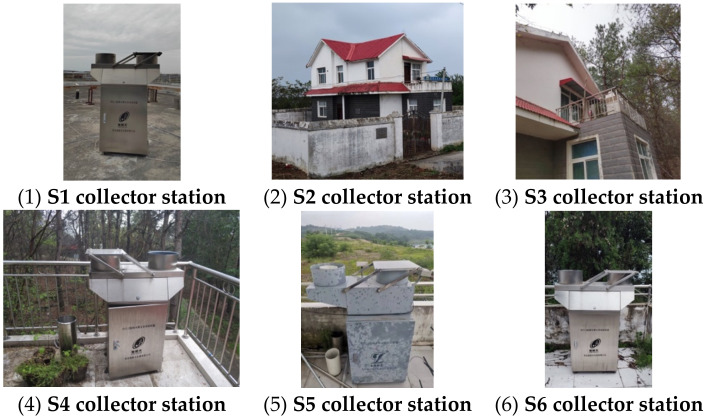
Nitrogen deposition sample collectors.

Dangzikou (S5) station is around the orange yard. Wulongquan (S6) station is in forest area, as shown in Figure 2.

### 2.2. Sampling Method

The automatic samplers were placed on the different functional areas, as shown in Figure 2. A sensor was installed on the sampler to control the collection of dry and wet deposition samples automatically. The machine will automatically open the cover to collect wet deposition once it rains. The cover will be automatically closed within five minutes after the precipitation.

Dry deposition samples are collected once a week and four times a month. Wet deposition samples are collected once in one rainfall. If there are multiple rainfalls a day, they shall be combined into one sample. The collected samples are placed in a brown polyethylene bottle, stored in a refrigerator at −20 °C, and the analysis is completed within one week. The dimension of the collector is length 1.2 m × width 0.8 m × height 1.5 m, consisting of a dust tank (d = 150 mm) and a rainfall funnel (d = 300 mm).

The dry and wet deposition fluxes of atmospheric nitrogen around the reservoir area are obtained by Formulas (1) and (2) [25].

Dry deposition flux

(1) $$F_{d}=\frac{K_{d}*C*V}{S_{d}}=0.5659*C*V$$

where *F_d_* is the monthly dry deposition flux, kg · hm^−2^ · month^−1^, *K_d_* is the conversion coefficient, 10^−2^, *C* is the nitrogen concentration in the collected solution, mg/L, *V* is the volume of collected liquid, L, *S_d_* is the area of dust cylinder, 0.0177 m^2^, and *K_d_*/*S_d_* = 0.5659 hm^−2^.

Wet deposition flux

(2) $$F_{w}=\sum_{i=1}^{n}\frac{K_{w}*C_{i}*V_{i}}{S_{w}}=\sum_{i=1}^{n}0.1415*C_{i}*V_{i}$$

where *F_w_* is the monthly wet deposition flux, kg · hm^−2^ · month^−1^, *K_w_* is the conversion coefficient, 10^−2^, *C_i_* is the nitrogen concentration in rainfall collected in one month, mg/L, *V_i_* is the volume of each rainfall, L, *S_w_* is the area of rainfall funnel, 0.0707 m^2^, and *K_w_*/*S_w_* = 0.1415 hm^−2^.

NO_3_—N was measured by phenol disulfonic acid spectrophotometry [26] (HJ/T346—2007), and the detection limit was 0.02–2.00 mg · L^−1^; NH_4_^+^-N was measured by Nessler reagent spectrophotometry [27] (HJ525—2009), and the detection limit was 0.025–2.00 mg · L^−1^. DTN was measured by potassium persulfate digestion ultraviolet spectrophotometry [28] (HJ636—2012), and the detection limit was 0.05–4.00 mg · L^−1^. Organic nitrogen = Total nitrogen—Tnorganic nitrogen (NO_3_—N + NH_4_^+^—N). Urea-N is determined by diacetyl MONOOXIME thiosemicarbazone spectrophotometry [29]. Shimadzu UV-2600 (Shimadzu Enterprise Management (China) Co., Ltd., Shanghai, China) was used to determine the absorbance at 525 nm and to calculate the urea content in the sample.

## 3. Results

The nitrogen deposition was detected from January 2019 to May 2020. In the two recent years before and after the COVID-19 lockdown, the DTN, DON, and Urea-N in the atmospheric nitrogen deposition was investigated.

### 3.1. DTN Distribution

In 2020, the DTN in the early lockdown period was 1.83 kg · hm^−2^ · month^−1^ (variation coefficient between stations was 0.399), and the monthly average DTN during the middle epidemic lockdown period was 1.99 kg · hm^−2^ · month^−1^ (variation coefficient 0.14–0.45), which is an increase of 8.7%. After the epidemic lockdown, the amount of DTN in May was 2.56 kg · hm^−2^ · month^−1^ (coefficient of variation 0.24), an increase of 28.41%. Compared with the same period in 2019, the average monthly DTN during the middle epidemic lockdown period decreased from 2.21 kg · hm^−2^ · month^−1^ to 1. 99 kg · hm^−2^ · month^−1^, a decrease of 9.6%.

**Figure 3:**
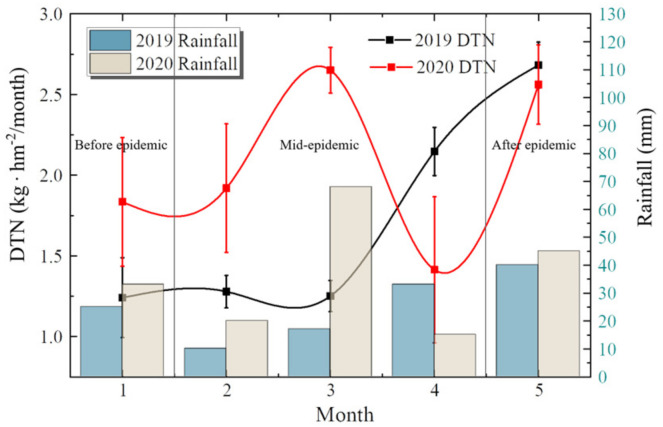
DTN around COVID-19 lockdown.

As shown in Figure 3, the total amount of DTN increased slightly during the middle epidemic period after the lockdown in 2020, but it was lower than that in the same period in 2019. After the lockdown of the city, the amount of DTN increased significantly, close to that in 2019.

By Pearson correlation coefficient, the correlation between DTN and rainfall is 88.66% in 2019 and 89.89% in 2020. The average rainfall from February to April 2019 is 20 mm, compared with 34 mm in 2020, which is one of the reasons for the rise of DTN during the epidemic in 2020.

### 3.2. DON Distribution

In 2020, the DON in the early lockdown period was 0.84 kg · hm^−2^ · month^−1^ (variation coefficient 0.329), and the monthly average DON during the middle epidemic lockdown period was 0.80 kg · hm^−2^ · month^−1^ (variation coefficient 0.19–0.56), a decrease of 4.8%, DON was 1.06 kg · hm^−2^ · month^−1^ (variation coefficient 0.25) in May. After the epidemic lockdown, DON increased by 31.86%. Compared with the same period in 2019, the average monthly DON during the middle epidemic period decreased from 1.15 kg · hm^−2^ · month^−1^ to 0.8 kg · hm^−2^ · month^−1^, a decrease of 30.4%.

The data shows that the total amount of DON decreased during the middle epidemic period, lower than that in the same period in 2019. After the lockdown, the amount of DON increased significantly, but it was also far lower than that in the same period in 2019.

**Figure 4:**
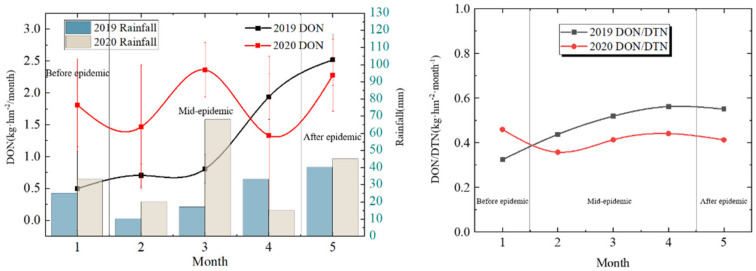
DON around COVID-19 lockdown.

As shown in Figure 4, in the early lockdown period, the DON/DTN was 0.46. During the middle epidemic lockdown period, the monthly average DON/DTN was 0.40, a decrease of 6%. After the epidemic lockdown finished at the end of April, the DON/DTN was 0.41. Compared with the same period in 2019, the average monthly DON/DTN during the middle epidemic period decreased from 0.51 to 0.4, a decrease of 11%. It shows that DON decreased slightly during the middle epidemic period, and the decline is larger than that in 2019.

### 3.3. Urea-N Distribution

Before the epidemic lockdown, the monthly Urea-N was 0.17 kg · hm^−2^ · month^−1^. During the middle epidemic lockdown period, the monthly average Urea-N was 0.15 kg · hm^−2^ · month^−1^, a decrease of 9.85%. After the lockdown was finished, the amount of Urea-N was 0.17 kg · hm^−2^ · month^−1^, up 9.3%. Compared with the same period in 2019, the average monthly Urea-N during the middle epidemic period decreased from 0.22 kg · hm^−2^ · month^−1^ to 0.15 kg · hm^−2^ · month^−1^, a decrease of 28.97%.

**Figure 5:**
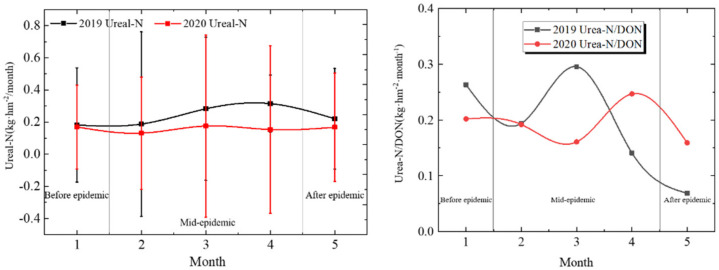
Urea-N around COVID-19 lockdown.

As shown in Figure 5, the total amount of Urea-N during the middle epidemic period is also less than that in the early/later epidemic lockdown periods and last year. It can be considered that human activities and agricultural activities have been affected by the epidemic lockdown.

In the early epidemic lockdown period, the Urea-N/DON of the current month was 0.20. During the middle lockdown period, the monthly average Urea-N/DON was 0.20. After the epidemic lockdown from the end of April, the Urea-N/DON was 0.16. Compared with the same period in 2019, the average monthly Urea-N/DON during the middle lockdown period decreased from 0.21 to 0.20, a decrease of 4.99%. It shows that during the middle lockdown period, the change of Urea-N/DON is smaller than that in the early/later lockdown periods and the same period in 2019.

Urea-N in the atmosphere comes from multiple sources such as agricultural activities, biomass combustion, excretion of final nitrogen products of animal metabolism, and decomposition of protein substances [30,31,32].

## 4. Discussion

### 4.1. Climatic Conditions of Reservoir

The Danjiangkou reservoir is located between Henan and Hubei province, which is the upstream of the Hanjiang River, a tributary of the Yangtze River. The reservoir location area is the north subtropical monsoon climate, which has the characteristics of sufficient precipitation, abundant heat and four distinct seasons. The annual sunshine number is 1950 h and the sunshine rate is 44%. Each square centimeter of land receives 104.8 kcal of radiant energy throughout the year. The average temperature is 15.6–16.0 °C, with an annual range of 24.7 °C. The highest temperature is in July, with an average temperature of 27.8 °C; The lowest temperature is January, and the average temperature is 3.1 °C. The frost-free period in the Danjiangkou reservoir area is 254 days. The average annual precipitation is 750–900 mm, and the interannual precipitation varies greatly. The precipitation in summer is 30–49% of the annual precipitation; Only 4–6% in winter; The precipitation in spring and autumn is similar, the dam elevation is 176.6 m, and the out flow is 500 m^3^/s.

### 4.2. Deposition Flux Comparison

The sources of DON, including industrial and agricultural activities, aquaculture and animal husbandry production, combustion of coal and biomass, waste treatment, direct volatilization of soil humus and plant pollen, animals and plants, and products of photochemical reaction between active NOx and hydrocarbons in the atmosphere, are complex and diverse.

Fenn et al. [33] shown the wet deposition (0.9–2.0 kg · hm^−1^ · yr^−1^) and through fall (0.5–1.2 kg · hm^−1^ · yr^−1^) deposition of inorganic N in the three national parks. The estimated total N deposition levels reported for the forest stands studied in the three parks (1.3–2.1 kg · hm^−1^ · yr^−1^). 

**Figure 6:**
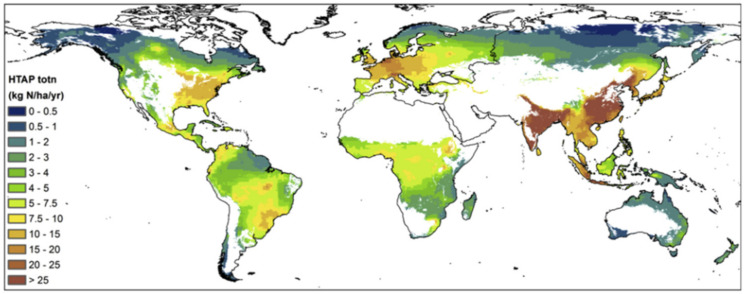
Multimodel mean total N deposition (kg·hm^−1^·yr^−1^) from the HTAP2 project on grid cells with a forest cover >5% (Donna B. Schwede, 2018).

Donna et al. [34] provide updated spatial distribution and inventory data for on-road NH_3_ emissions for the continental United States (U.S.) On-road NH_3_ emissions were determined from on-road CO_2_ emissions data and empirical NH_3_: CO_2_ vehicle emissions ratios. Emissions of NH_3_ from on-road sources in urbanized regions are typically 0.1–1.3 t · km^−2^ · yr^−1^, while NH_3_ emissions in agricultural regions generally range from 0.4–5.5 t· km^−2^ · yr^−1^, with a few hotspots as high as 5.5–11.2 t · km^−2^ · yr^−1^, as shown in Figure 6.

Total deposition of NHx-N, NOy-N and total N on the world’s terrestrial ecosystems for the year 2010. The Total N was up to 70.3 (Tg · N · yr^−1^) from Land use class of Forest, semi natural vegetation/grassland, Croplands and Other land [35,36,37].

The total N deposition was estimated at 80–100 kg N · ha^−1^ · yr^−1^ for a maize-wheat cropping system in the North China Plain, China (He et al., 2007, 2010 [38]), and 29.3 kg N · ha^−1^ · yr^−1^ in coastal sage scrub ecosystems in Riverside, California (Sickman et al.) [39].

Total N deposition of 43 sites was ranked by land use as urban > rural > background sites (Xu et al) [40].

Xiao et al. [41] reported that the total N deposition rates were 13.8–47.7 kg · hm^−1^ · yr^−1^ at five sites in Yangtze River Basin, China.

The data of European Monito-ring and Evaluation Programme (EMEP, 2003–2005) shows that the nitrogen wet deposition flux in Europe from 2003 to 2005 is 1.04–18.4 kg · hm^−2^ · a^−1^, The monitoring results in this work are obviously higher than the above research data.

It can be seen the nitrogen deposition flux in this region is at a medium level in China, but much higher than that in remote areas with less human interference, indicating that the nitrogen deposition is greatly affected by human activities.

The areas with high concentration in the epidemic situation have decreased significantly, and the areas with high deposition have been transferred to the orchard and tourism areas. The forest area belongs to deciduous broad-leaved forest, and its active nitrogen emission mainly comes from soil organic matter, animal and plant decay, plant allelochemicals, and pollen particles.

### 4.3. Effects of Land Use Types on Nitrogen Deposition

#### 4.3.1. Analysis of Land Use Types

**Figure 7:**
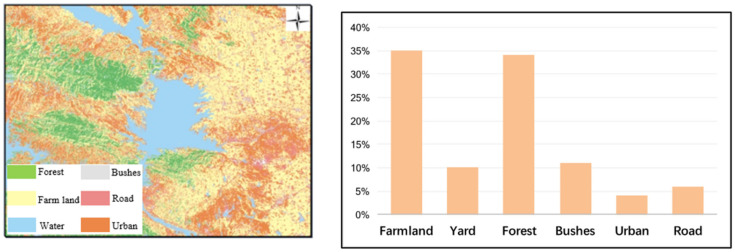
Land types in the Danjiangkou Reservoir Area.

The land around Xichuan reservoir area is mainly farmland and forest. The farmland is 45% (including about 10% orange yard), mostly distributed in hilly areas, with 34% of forest area, mostly distributed in mountainous areas, followed by grassland and bushes area, accounting for about 11%, and urban area and traffic area accounting for about 10%. The East and North banks of the reservoir are mainly agricultural area, and the West Bank is mainly forest area, as shown in Figure 7.

#### 4.3.2. Distribution of DTN before and after the Epidemic

In 2020, before the epidemic lockdown, the maximum value of DTN was at S4 station, 3.12 kg · hm^−2^/month, followed by S1 station, 2.24 kg · hm^−2^/month, and the minimum value was at S5 station, 0.866 kg · hm^−2^/month. The variation coefficient of between the six stations was 0.398.

In the middle epidemic lockdown period, the average maximum value of DTN was at S1 station, 2.67 kg · hm^−2^/month, followed by S4 station, 2.26 kg · hm^−2^/month, and the minimum value was at S6 station, 1.32 kg · hm^−2^/month. The maximum value of DTN decreased and transferred from traffic area to urban area. The variation coefficient between the six stations decreased, indicating that the variation of DTN in each region decreased.

After the epidemic lockdown period, the maximum DTN was at S1 station, 2.72 kg · hm^−2^/month, followed by S4 station, 2.33 kg · hm^−2^/month, and the minimum value was at S6 station, 1.43 kg · hm^−2^/month, with a variation coefficient of 0.12. The DTN increased after lockdown finish and the highest place is still in urban area.

**Figure 8:**
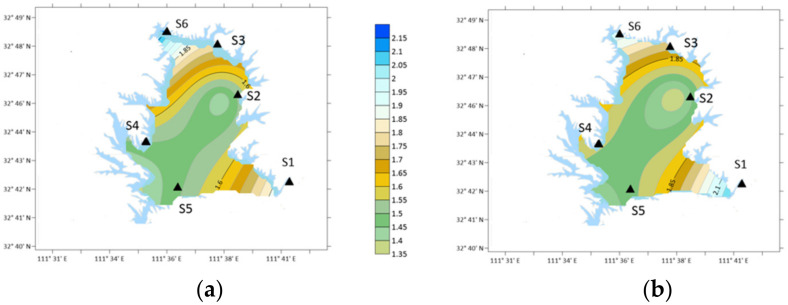

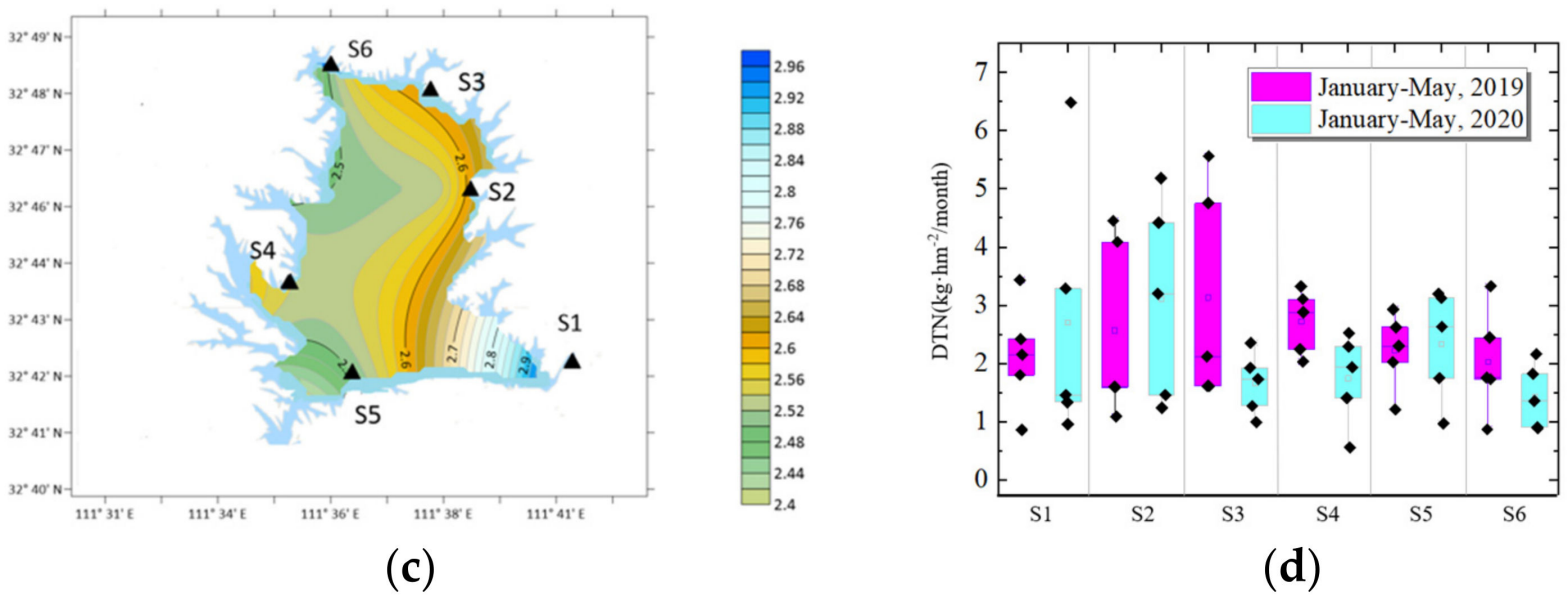
DTN distribution around COVID-19 lockdown. (**a**) Before lockdown; (**b**) Middle lockdown; (**c**) After lockdown; (**d**) DTN compared with 2019.

As shown in Figure 8, the data in S5 and S2 station decreased more significantly in the 2020 epidemic period than in the same period in 2019. S5 decreased from 3.13 kg · hm^−2^/month to 2.03 kg · hm^−2^/month, and S2 decreased from 2.70 kg · hm^−2^/month to 1.74 kg · hm^−2^/month, a decreased of 1 kg · hm^−2^/month. S5 is yard area and S2 is traffic area.

#### 4.3.3. Distribution of DON before and after the Epidemic

In the early lockdown period in 2020, the maximum value of DON was at S4 station, 1.37 kg · hm^−2^/month, followed by S3 station, 0.98 kg · hm^−2^/month, and the minimum value was at S1 station, 0.49 kg · hm^−2^/month. The variation coefficient between the six stations was 0.329.

In the middle lockdown period, in 2020, the average maximum value of DON was at S5 station, 1.09 kg · hm^−2^/month, followed by S3 station, 0.95 kg · hm^−2^/month, and the minimum value was at S6 station, 0.49 kg · hm^−2^/month. The variation coefficient of six locations was 0.257. The maximum value of DON decreased and shifted from traffic area to yard area. The locations variation coefficient decreased, indicating that the change of DTN in each region decreased.

After the epidemic lockdown, the maximum value was at S1 staiton, 1.55 kg · hm^−2^/month, followed by S5 station, 1.14 kg · hm^−2^/month, the minimum value was at S6 station, 0.69 kg · hm^−2^/month, and the variation coefficient was 0.258. The DON value increased after the lockdown finished, and the highest values occurred in urban areas.

**Figure 9:**
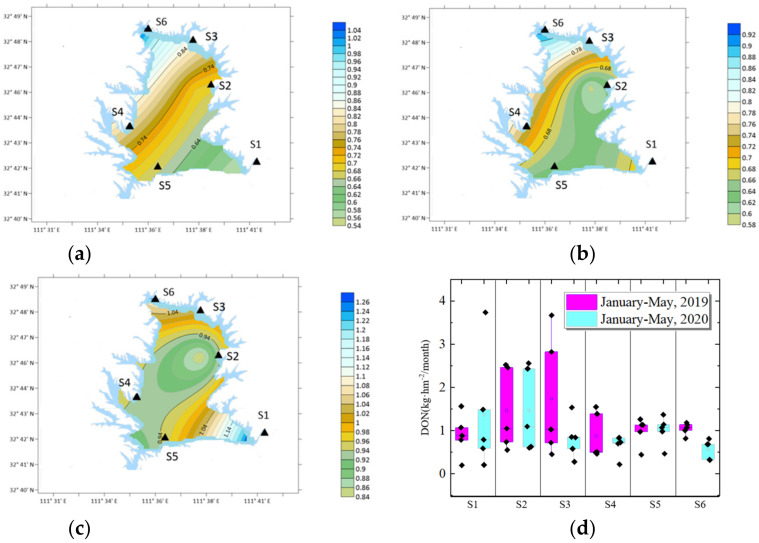
DON distribution around COVID-19 lockdown. (**a**) Before lockdown; (**b**) middle lockdown; (**c**) after lockdown; (**d**) DON compared with 2019.

As shown in Figure 9, the average DON quantity in S2 and S5 decreased more significantly in the 2020 epidemic period than in 2019. The S2 data decreased from 1.36 kg · hm^−2^/month to 0.66 kg · hm^−2^/month, and S5 data decreased from 1.74 kg · hm^−2^/month to 1.05 kg · hm^−2^/month, a decrease of 0.7 kg · hm^−2^/month, respectively.

S2 refers to traffic area; the lockdown activity results in nearly zero traffic.

S5 location is yard area; DON deposition mainly comes from agricultural activities and fossil fuel combustion. Around S5, the station is surrounded by a large orange yard. Germination fertilizer is applied in February, pre-flowering fertilizer is applied in March, and stable fruit fertilizer is applied in May. Plants and soil humus directly volatilize a large quantity of DON into the atmosphere, resulting in a large quantity of DON deposition.

Due to the epidemic lockdown, the orange fertilization, land tillage, wheat fertilization and travel in the city cannot be carried out, so nitrogen deposition is reduced.

#### 4.3.4. Distribution of Urea-N before and after the Epidemic

In early lockdown period, the maximum value of Urea-N was at S3 station, 0.215 kg · hm^−2^/month, followed by S3, 0.20 kg · hm^−2^/month, and the minimum value was at S4 station, 0.07 kg · hm^−2^/month. The variation coefficient between the six stations was 0.26.

In the middle lockdown period, the average maximum value of Urea-N was S1, 0.25 kg · hm^−2^/month, followed by S3, 0.22 kg · hm^−2^/month, and the minimum value was S4 yard, 0.07 kg · hm^−2^/month. The variation coefficient of six locations was 0.41. The maximum value of Urea-N decreased and shifted from agricultural area to urban area. The variation coefficient increased, indicating that the Urea-N in each region was quite different.

After the epidemic, the maximum value was at S1, 0.23 kg · hm^−2^/month, followed by S6, 0.23 kg · hm^−2^/month, and the minimum value was S6, 0.69 kg · hm^−2^/month, with a variation coefficient of 0.34. the Urea-N increased after lockdown and the highest place is still in urban area.

**Figure 10:**
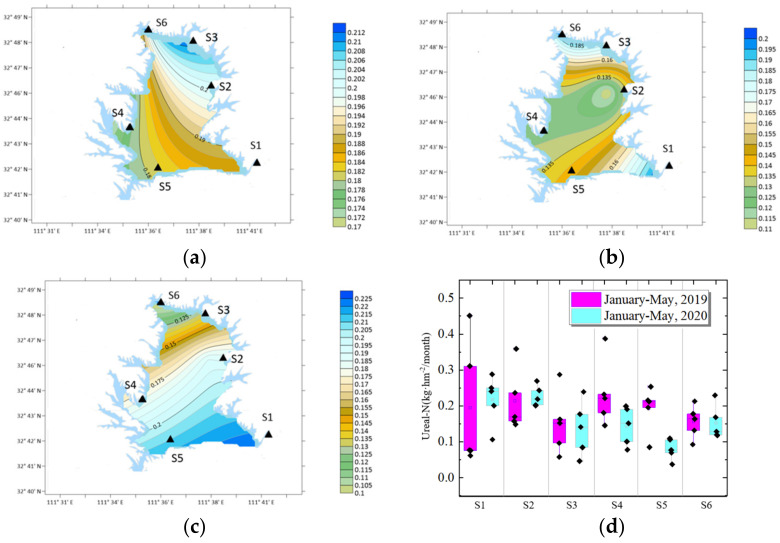
Urea-N distribution around COVID-19 lockdown. (**a**) Before lockdown; (**b**) middle lockdown; (**c**) after lockdown; (**d**) Urea-N compared with 2019.

It can be seen from Figure 10 that compared with the same period in 2019, the average amount of Urea-N in S4 area decreased significantly from 0.23 kg · hm^−2^/month to 0.080 kg · hm^−2^/month.

S4 Daguanyuan is a traffic area, indicating that traffic travel has been significantly affected by lockdown. Berman et al. [42] studied NO_2_ and PM2 during the US epidemic lockdown period and found that the concentration decreased in cities but did not change significantly in countryside areas.

### 4.4. Intra City and Intercity Travel during Lockdown

#### 4.4.1. Intra City Travel

**Figure 11:**
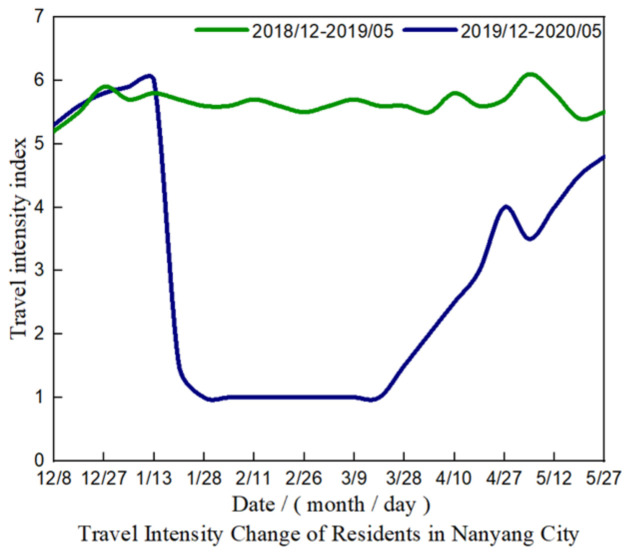
Intra city travel in lockdown period.

Urban travel intensity refers to the ratio of the population traveling in the city to the population living in the city. S4 is Daguanyuan, the main scenic spot in the city, and S2 is Songgang, the main wharf. It is the main destination and transit place for travel in the city. The impact of the epidemic lockdown has caused a sharp reduction in the intensity of urban wind travel, and the traffic area in the two places has a great impact. As shown in Figure 11, the Urban travel intensity in Nanyang city is reduced from 6 to 1 during the lockdown period.

#### 4.4.2. Intercity Travel

**Figure 12:**
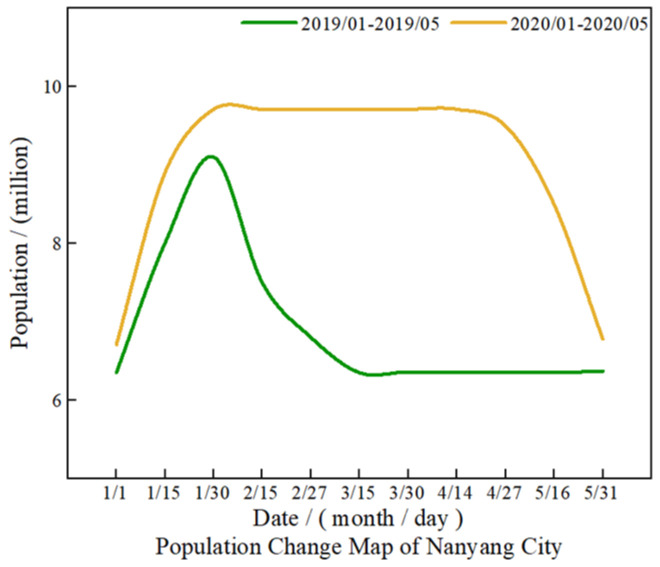
Urban population change.

Spring Festival transportation is a major migration of population with Chinese characteristics. China’s annual traffic reaches 2–3 billion people in 40 days. Nanyang is a city where population outflows, which means a large population work in southern China and back home during Spring Festival. The legal holiday of Spring Festival in China is 7 days. The returning population began to leave their hometown 6 days after the festival day. After the epidemic lockdown in January 2020, a large number of people stayed in their hometown. According to the statistics of Nanyang City Government, the permanent resident population of Nanyang in 2019 was 10.03 million, including 3.1 million people returning home during the Spring Festival, as shown in Figure 12.

As shown in Figure 12, during the 2019 Spring Festival transportation period, the Nanyang city’s railways, highway, and airports sent about 11.82 million person-time, and railway and road transportation are the main travel type 913 thousand passengers were sent by train. In terms of road transportation, the average daily transportation capacity of our city reaches 3388 vehicles, sending about 270,000 person-time per day and completing 10.8 million passengers per day. Nanyang Airport transported more than 113 thousand person-time and operated 1071 flights.

After the lockdown in 2020, the traffic flow within three months is almost zero. Therefore, nitrogen emissions have been significantly reduced.

## 5. Conclusions

(1) In December 2019, the COVID-19 outbreak broke out worldwide. On 30 January 2020, China had entered a state of comprehensive and strict control, and population movement was in lockdown. The periods of lockdown in China can be identified as three stages, the early epidemic period (January 2020), the middle epidemic period (February–April 2020), and the later epidemic period (May 2020). The impact of human activities on AND was investigated in January to May in 2019 and 2020.
(2) The Danjiangkou reservoir is the source of the middle route of South-to-North Water Diversion Project in China. The water quality of the Danjiangkou reservoir is directly related to the water safety of more than 20 large cities, such as Beijing and Tianjin. The AND has become a risk to the reservoir water quality.
(3) The lockdown activity resulted in a decrease of DTN, DON and Urea-N from the atmosphere in February to April 2020, decreasing by 9.6%, 30.4%, and 28.97%, respectively, compared to 2019. During the middle lockdown period, the DON decreased from 0.84 kg · hm−2·month−1 to 0.80 kg · hm−2 · month−1, and the Urea-N decreased from 0.17 kg·hm−2 · month−1 to 0.15 kg · hm−2 · month−1.
(4) The nitrogen deposition flux in this region is at a medium level in China, but it is much higher than that in remote areas with less human interference, indicating that the nitrogen deposition is greatly affected by human activities.
(5) The decrease can be explained by traffic stopping and fertilization being delayed, with the peak values of detected data between positions occurring in urban area and farmland. Necessary fertilization activities shall be carried out according to the growth cycle of crops, especially the addition of nitrogen-containing fertilizer, which has a significant impact on atmospheric Urea-N deposition.
